# Cognitive dysfunction and its association with inflammation in acute exacerbations of COPD: protocol for a prospective hospital-based cohort

**DOI:** 10.1136/bmjopen-2025-108239

**Published:** 2025-10-23

**Authors:** Simone N De Luca, Louise M Burrell, Allison Collins, Melinda Jackson, Ross Vlahos, Christine F McDonald

**Affiliations:** 1 Centre for Respiratory Science & Health, School of Health and Biomedical Sciences, RMIT University, Melbourne, Victoria, Australia; 2 Institute for Breathing and Sleep, Heidelberg, Victoria, Australia; 3 Department of Medicine, Austin Health, University of Melbourne, Melbourne, Victoria, Australia; 4 School of Psychological Sciences, Turner Institute for Brain & Mental Health, Melbourne, Victoria, Australia; 5 Department of Respiratory and Sleep Medicine, Austin Health, Heidelberg, Victoria, Australia

**Keywords:** Inflammation, Emphysema, Cognitive dysfunction

## Abstract

**Abstract:**

**Introduction:**

Chronic obstructive pulmonary disease (COPD) is characterised by progressive airflow limitation that is not fully reversible and is associated with an abnormal inflammatory response of lungs to noxious particles and gases. The inflammatory and reparative processes occurring in the lungs induce a ‘spill-over’ of inflammatory mediators into the circulation, resulting in an increase in systemic inflammation, which is further increased during acute exacerbations of COPD (AECOPD), leading to the development of extra-pulmonary comorbidities, such as cognitive impairment. Cognitive impairment affects up to 61% of people living with COPD. Heightened levels of inflammation have been linked to increased risk of cognitive impairments; however, the exact mechanisms remain unclear, thus hampering the development of therapeutics. This study aims to determine whether patients hospitalised with an acute COPD exacerbation show impaired cognitive function compared with recovery (~day 45), and whether such dysfunction is associated with systemic inflammation and oxidative stress.

**Methods and analysis:**

A prospective, observational study will be conducted at Austin Health in Victoria, Australia. Eligible participants will be assessed during admission for AECOPD and following stabilisation (approximately day 45). The primary outcome is the difference in cognitive function between admission for AECOPD to recovery using non-verbal cognitive tests. Secondary outcomes are changes in systemic markers of inflammation, oxidative stress and ACE2 catalytic activity. Tertiary outcomes are anxiety and depression scores.

**Ethics and dissemination:**

Ethical approval has been granted in Australia by Austin Health Human Research Ethics Committee (HREC 56099) with governance approval at Austin Hospital. The results will be published in peer-reviewed scientific journals and presented at national and international scientific conferences. Findings will be disseminated to consumers in publications for lay audiences.

**STRENGTHS AND LIMITATIONS OF THIS STUDY**

- This study uses precise, sensitive and objective computerised cognitive testing (Cambridge Cognition - CANTAB (Cambridge Neuropsychological Test Automated Battery)) alongside Mini Mental State Examination to assess multiple cognitive domains.
- The CANTAB platform is culturally neutral, non-invasive and non-verbal, reducing language and literacy bias in assessment.
- This study will assess systemic inflammation, oxidative stress and ACE2 in parallel with cognitive testing, enabling evaluation of biomarker inter-relationships with neuropsychological outcomes in chronic obstructive pulmonary disease exacerbations.
- A limitation is the relatively small sample size, which may reduce power to detect modest associations, particularly in exploratory correlation analyses.

## Introduction

 Chronic obstructive pulmonary disease (COPD) is a chronic and progressive lung disease that is characterised by an irreversible reduction in airflow and is associated with abnormal lung inflammation in response to cumulative gene-environment interactions over a lifetime.^1^ COPD has a similar prevalence in men and women, and while cigarette smoking is the major cause of COPD in industrialised countries,^1^ an estimated 10.5% of Australians with COPD are never-smokers.^2^ Individuals living with COPD frequently experience heightened airway dysfunction and respiratory symptoms often caused by viral and bacterial infections. The average person with COPD will experience two exacerbations per year, with 10% of these episodes requiring hospitalisation and an increased in-patient mortality rate.^3 4^

COPD leads to airway obstruction which is commonly associated with increased inflammation in the airways and bronchoalveolar lavage fluid characterised by significant rises in macrophages and neutrophils and elevated levels of pro-inflammatory cytokines such as tumour necrosis factor-α (TNF-α) and interleukin (IL)-1β.^5^ Additionally, there is increased oxidative stress from reactive oxygen species (ROS) and reactive nitrogen species, along with a reduction in antioxidants (eg, glutathione peroxidase, catalase and superoxide dismutase) in both the airways and lung parenchyma.^6^ This persistent low-grade inflammation causes the destruction of alveolar walls and excessive mucus secretion, leading to structural damage in both small and large airways (bronchitis) and lung parenchyma (emphysema).^7^ Importantly, literature has shown that the inflammatory and reparatory events that occur in the lungs of both stable and exacerbated COPD patients may ‘spill-over’ into the circulation, resulting in the manifestation of extra-pulmonary comorbidities including cognitive impairment.^8^,^12^ The presence of cognitive impairment greatly potentiates the morbidity and mortality of individuals with COPD and their related healthcare costs.

Individuals with COPD are at a significantly higher risk (61%) of developing impairment in areas such as memory, executive function and attention compared with age-matched healthy individuals (12%).^13^,^16^ Moreover, people with COPD also have an increased risk of developing neurodegenerative diseases, with, for example, a 74% greater likelihood of developing Alzheimer’s disease compared with age-matched and gender-matched controls.^17^ During an acute exacerbation of COPD (AECOPD) event, individuals commonly experience a decline in cognitive function. The recovery time for cognitive function after an exacerbation is unclear and many individuals may not fully recover.^13 14^ There are no therapies capable of slowing or preventing accelerated cognitive dysfunction in COPD as the cellular and molecular mechanisms that drive susceptibility are not well characterised.

Cognitive impairment associated with COPD has been linked to increased ROS in individuals living with COPD^18^ and chronic hypoxaemia.^19^ Cognitive dysfunction is a known consequence of cerebral small-vessel disease and recent neuroimaging studies suggest that occult cerebrovascular damage plays a key role in brain damage and dysfunction (ie, alterations in grey and white matter and cerebral atrophy) in COPD.^20^,^24^ It is well established that oxidative stress can alter cerebral vessels, leading to vascular remodelling, stiffness, atherosclerosis and blood-brain barrier disruption.^25^ Additionally, pro-inflammatory cytokines such as TNF-α and IL-6 alter the functioning of cerebral vessels by increasing ROS production.^26 27^ ROS can directly promote inflammation in the vessel wall by inducing the production of cytokines and pro-inflammatory genes.^28^ We have shown that markers of oxidative stress and inflammation (serum amyloid A (SAA) and C-reactive protein (CRP)) are increased in human lungs and blood during an exacerbation of COPD.^29^,^31^ Thus, increased oxidative stress and systemic inflammation in COPD may exacerbate cerebral blood vessel damage, leading to cognitive impairments in COPD. Understanding the underlying mechanisms is crucial for primary prevention (reduce the risk of developing cognitive impairments and dementia) and early intervention (slow the progression or development of cognitive impairments and dementia) in individuals with COPD.

The aim of this study is to determine whether patients admitted to hospital with an acute exacerbation of COPD have impaired cognitive function compared with after recovery at approximately day 45 and whether this observed cognitive dysfunction is associated with increased systemic inflammation, oxidative stress and peripheral biomarkers of cognitive dysfunction. We hypothesise that (1) pulmonary inflammation and oxidative stress that ‘spill-over’ from the lungs into the central nervous system are driving brain inflammation and cognitive impairments and (2) AECOPD participants will have increased systemic inflammation and thus cognitive impairment compared with the same participants assessed in the stable state after recovery.

## Methods and analysis

### Design

**Figure 1:**
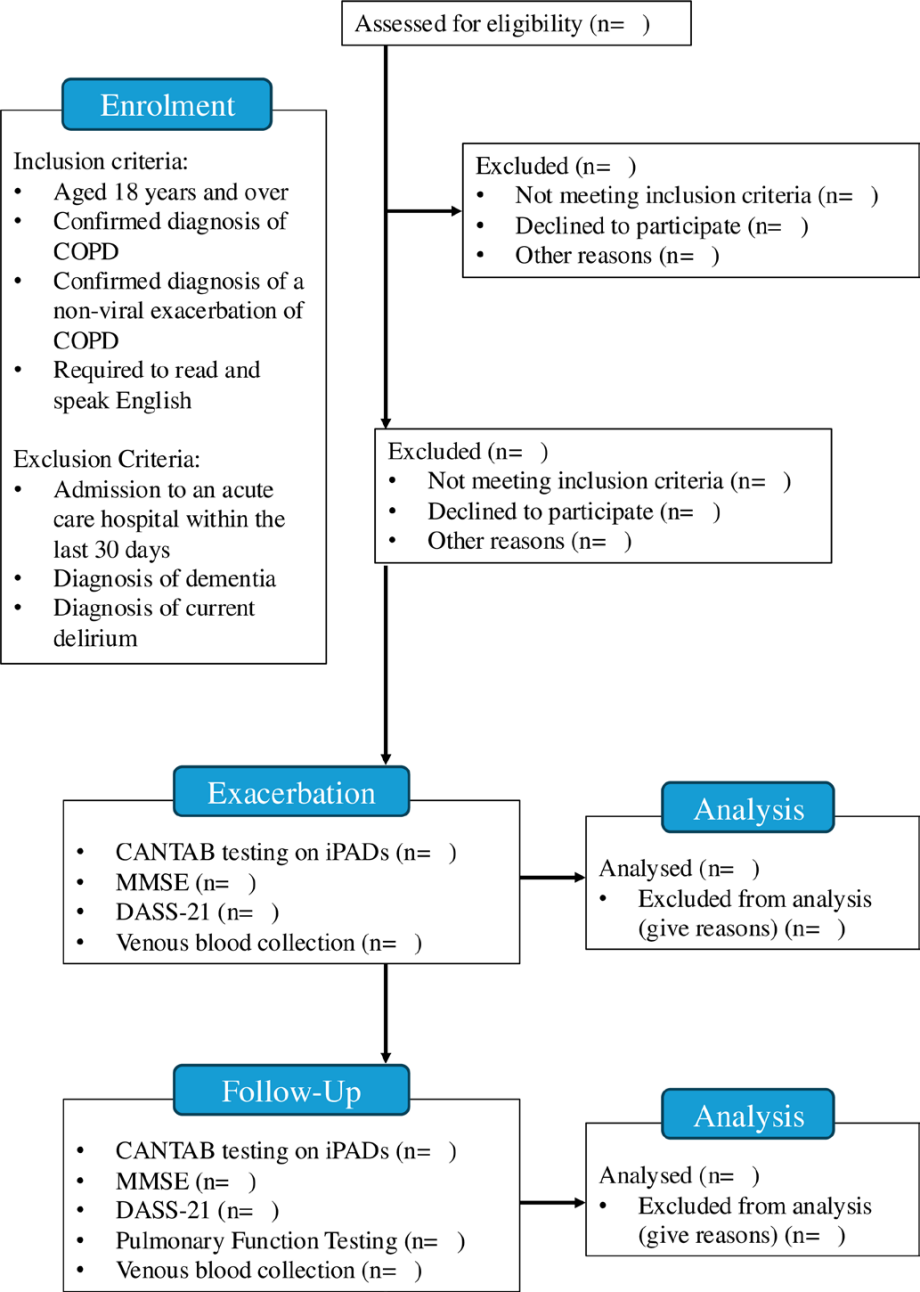
Participant flow through the study. COPD: Chronic obstructive Pulmonary Disease. CANTAB: Cambridge Neuropsychological Test Automated Battery. MMSE: Mini Mental State Examiniation. DASS-21: Depression, Anxiety and Stress Scale - 21 Items.

A prospective, observational cohort study will be conducted at Austin Health, in Heidelberg, Australia. Figure 1 shows the participant flow through the study.

### Patient and public involvement

Patients and members of the public were not involved in the design, conduct, reporting or dissemination plans of this research.

### Participants

People who have been hospitalised for an exacerbation of COPD at the Austin Hospital will be invited to participate. Patients will be eligible for inclusion if they (1) are aged 18 years and over, (2) have a confirmed diagnosis of COPD, (3) swab negative for common respiratory viruses and (4) are able to read and speak English. Participants will be excluded if they (1) have been admitted to an acute care hospital within the last 30 days for an exacerbation of COPD and (2) have had a previous diagnosis of dementia or of current delirium (table 1).

**Table 1:** Assessment schedule

Assessment/procedure	Enrolment	Acute exacerbations of chronic obstructive pulmonary disease	Stabilisation
Informed consent	x		
Cognitive testing via CANTAB (Cambridge Neuropsychological Test Automated Battery) Connect		x	x
Mini Mental State Examination		x	x
Depression Anxiety Stress Scale Questionnaire		x	x
Cognitive Failure Questionnaire		x	x
Venous blood collection		x	x
Resting arterial blood gas			x
Respiratory function testing			x
Adverse events		x	x

### Recruitment

Potential participants admitted with a non-infective exacerbation of COPD or following clearance from a viral exacerbation (excluding COVID-19) will first be identified by the respiratory ward medical team and provided with a patient information sheet. If the participant is interested in obtaining more detailed information, the clinical trials’ nurse and principal investigator will then address any questions the patient may have regarding participation prior to obtaining written informed consent, who is not in a dependent relationship with the patient and will provide further information. Participation is voluntary and participants will not be offered any inducements to take part in the study. The study coordinator will inform the patients that their decision to participate or not will not affect their care, that their data will be held securely and that they will not be identified in any study publication. Written informed consent will be obtained by study investigators once participants have had adequate opportunity to consider their involvement and ask questions. Recruitment commenced in September 2022 and recruitment will cease in December 2025.

### Safety considerations

There are no specific safety considerations associated with the study.

### Procedure

Outcome measures will be collected during admission for AECOPD and following stabilisation in the outpatient setting (approximately day 45). Day 45 was selected as participants will likely be in a stable state after recovery from the exacerbation event. Venous blood samples will be drawn once during admission for AECOPD and once during recovery (approximately day 45) and centrifuged to collect platelet-poor plasma.

### Outcome measures

The primary outcome is the change in cognitive function (measured by Cambridge Cognition - CANTAB Connect and conventional MMSE testing) during admission for AECOPD and upon recovery at approximately day 45. Secondary outcomes include changes in markers of systemic inflammation (CRP, SAA, IL-6, TNF-α), oxidative stress markers (8-isoprostane, thiobarbituric acid reactive substances), ACE 2 and the cognitive function marker, clusterin. Tertiary outcomes will include anxiety and depression evaluation using the Depression Anxiety Stress Scale Questionnaire (DASS-21) and self-reported perception, memory and motor lapses in daily life using the Cognitive Failure Questionnaire (CFQ).

### Data collection

#### Cognitive function

Key cognitive domains suggested to be impaired in COPD individuals will be assessed using precise, highly sensitive and objective state-of-the-art computerised cognitive testing.^24 32^ Cambridge Cognition CANTAB (Cambridge Neuropsychological Test Automated Battery) Connect is a culturally neutral, non-invasive and non-verbal cognitive assessment that does not require technical expertise, making it suitable for diverse populations. Responses are automatically captured and scored. We will first assess the participants’ motor response speed (CANTAB Motor Screening task), processing speed (CANTAB Reaction Time), ability to perform the object location associative memory (CANTAB PAL) and attention and working memory (CANTAB Spatial Working Memory). The MMSE screening test will be performed for comparison to the current literature.^24 32^ The CANTAB Connect tests will be administered by iPAD, and the MMSE will be administered by conventional face-to-face procedure by a trained research assistant.

#### Anxiety and depression status

Anxiety and depression status will be measured at time-points using the paper-based version of the DASS-21 and CFQ.

#### Blood biomarkers

For serum biomarker analysis, venous blood will be collected into serum-separating tubes (BD Vacutainer Serum Tubes), allowed to clot at room temperature for 60 min, and centrifuged at 1300×g for 10 min at room temperature. Serum will be aliquoted immediately into aliquots of approximately 250 µL. To prevent degradation of isoprostanes, 5 mg/mL butylhydroxytoluene (Sigma-Aldrich) will be added to a single aliquot. Aliquots will then be stored at –80°C to minimise degradation and eliminate repeated freeze–thaw cycles. These procedures are consistent with best practice for cytokine and inflammatory marker preservation. Samples will be batch processed to avoid inter-assay variability in results.

Measurements will be performed blinded and independently. CRP will be assessed using high-sensitivity ELISA (detection limit 0.35 ng/mL; Alpha Diagnostics). IL-6 will be measured using a high-sensitivity ELISA (detection limit 4.7 pg/mL; BD OptEIA ELISA). TNF-α using a high-sensitivity ELISA (detection limit 15.6 pg/mL; Invitrogen) will be used to determine SAA (detection limit 1.1 ng/mL; Anogen). Malondialdehyde will be measured spectrophotometrically (Cell Biolabs). Isoprostane will be measured using a high-sensitivity ELISA (detection limit 1 pg/mL; Ray Biotech). All biomarkers are validated in serum and widely applied in large COPD and cognitive cohorts. Endogenous inhibitors of ACE2 activity will be removed and ACE2 catalytic activity will be measured using a validated, sensitive quenched fluorescent substrate-based assay.^33^ All samples will be assayed in duplicate with internal controls and pooled quality control samples on each plate to ensure reproducibility.

### Sample size

Our primary outcome is cognitive differences between AECOPD and recovery. Based on prior studies reporting moderate effect size in this population,^13^ a priori analysis was conducted in G*Power V.3.1.9.7 to determine the sample size required to sufficiently power the analysis. This requires ~30 participants to detect the expected within-subject change accounting for a standardised mean change of 0.6 with 90% power and alpha 0.05. To account for a 20% dropout, we plan to recruit 40 participants (target analysed n=32), which will be sufficient to detect the expected within-group change. Analysis of correlations between inflammatory/oxidative markers and cognitive change are considered secondary outcomes and will be reported with 95% CIs.

### Statistical analysis plan

Paired analyses will be conducted to compare cognitive outcomes and serum measures of systemic inflammation/oxidative stress between the acute AECOPD state (admission) and the recovered state (day 45). Paired t-tests (or Wilcoxon signed-rank tests if assumptions are not met) will provide the primary within-subject comparisons. To adjust for covariates, linear regression models will be employed with time (admission and recovery) as a fixed effect and participant identification included as a clustering variable, allowing the incorporation of covariates such as age, sex and smoking status. Log transformations will be applied for serum biomarkers, and sensitivity analyses will compare transformed versus untransformed results. All statistical tests will be two-sided with α=0.05, and effect sizes will be reported as Cohen’s d with 95% CIs.

The primary outcome will be cognitive change assessed by the MMSE. The secondary outcomes will include the CABTAB cognitive tasks and serum biomarkers. Initially, to test the hypothesis, models will be run with time (acute vs stable) as the only within-subject factor; additional models will then include covariates such as age or sex to examine potential moderation effects.

### Data integrity and management

Databases created as part of this project will contain identifiable participant information and deidentified research outcome data. The deidentified data can be reidentified using a participant code that is linked to the identifiable participant information. Electronic data will be stored in a purpose-built online database (www.redcap.com), which features encryption and password protection. The principal investigator and the study coordinator will have access to the electronic data. Additionally, any hard copies of participant identifying information will be kept in a locked cabinet within a secure facility at Austin Hospital. All biological samples will be collected according to recommended laboratory practices. Information will be stored indefinitely, in compliance with Human Research Ethics Committees requirements for observational studies.

## Ethics and dissemination

This study has received ethics approval in Australia from Austin Health Human Research Ethics Committee (HREC 56099) with governance approval at Austin Hospital. Any amendments to the study protocol will be reported to the Ethics Committee. The study will be conducted and reported according to the Standard Protocol Items: Recommendations for Interventional Trials guidelines and the Consolidated Standards of Reporting Trials statement. The study commenced in September 2022 and is currently recruiting participants. Prior to the commencement, participants will provide written informed consent. Study findings will be published in peer-reviewed journals and presented at national and international scientific meetings. We will also disseminate our findings to people living with COPD through lay publications and seminars.
