# Supplementary material for: Cognitive dysfunction and its association with inflammation in acute exacerbations of COPD: protocol for a prospective hospital-based cohort
Source: BMJ Open. 2025 Oct 23;15(10):e108239. doi: 10.1136/bmjopen-2025-108239 (PMC12557734; doi:10.1136/bmjopen-2025-108239)
Supplement: Supplementary file 1 [file bmjopen-2025-108239.reviewer_comments.pdf]

PEER REVIEW HISTORY

BMJ Open publishes all reviews undertaken for accepted manuscripts. Reviewers are asked to complete a checklist review form and are provided with free text boxes to elaborate on their assessment. These free text comments are reproduced below.

ARTICLE DETAILS

Title (Provisional)

Cognitive Dysfunction and its Association with Inflammation in Acute Exacerbations of COPD: Protocol for a Prospective Hospital-Based Cohort

Authors

De Luca, Simone; Burrell, Louise; Collins, Allison; Jackson, Melinda; Vlahos, Ross; McDonald, Christine F.

VERSION 1 - REVIEW

**Reviewer**	**1**
**Name**	**Clouston, Sean**
**Affiliation**	**Stony Brook University, Family, Population, and Preventive Medicine**
**Date**	**16-Sep-2025**
**COI**	**None**

The Blood Biomarkers proposed are not well described, I think. The measures section does not clarify if measures will be reported in both plasma and serum, but these can have somewhat different success rates. Notably, IL-6 and CRP can be fairly unstable and small biomarker measurement protocol variations could cause results to be uninterpretable. It would be good to be clear about the collection and storage protocols.

Typo in the abstract: "An prospective..." should be "A prospective..."

I found the power analysis to be confusing and maybe optimistic. In our data, for example, the correlation between CRP and IL-6 is R=0.33. A back of the napkin calculation suggests that a correlation of 0.33 is powered (using power = 0.9, a = 0.05) only in a sample with ≥95 people. It would be good for the authors to explain their expected effect sizes and what their power simulations assumed.

I don't know what this means: "Outcome measures will be collected during admission for AECOPD and following stabilization in the outpatient setting (approximately day 45). Day 45 was selected as participants will likely be in a stable state after recovery from the exacerbation event." I think this means that the authors will have two time-points at study

entry and at day 45? If so, the multilevel models cannot be appropriately run and a third time-point would be expected.

For recruitment, the authors note that "Potential participants will be identified by their treating healthcare team. If the participant is interested in obtaining more detailed information, they will be contacted by the study coordinator, who is not in a dependent relationship with the patient and will provide further information." I am not sure who judges whether the participant is interested, nor what the discussion around personal benefits to participating in research could be in the Australian situation. A lack of understanding about the incentives drawing individuals into your study could reduce generalizability of any findings so some discussion of this process could be helpful.

ACE2 is highly associated with COVID-19 severity and, therefore, with the presence of post-acute sequelae of COVID-19 (long-COVID or PASC). Since studies suggest that there are changes in cognition aligning directly with PASC as well, it would be good for the authors to include a COVID infection history and a PASC symptoms inventory.

Linear mixed methods are proposed for this study. These are likely to work well with some of the outcomes, but may not work well with the MMSE (which is highly abnormally distributed in most populations) and could have difficulty with some of the biomarkers (which tend to be skewed away from a their respective biological floors). The authors might consider secondary approaches in the case that they find that the longitudinal variance is abnormal.

VERSION 1 - AUTHOR RESPONSE

Reviewer 1

Comment 1: The Blood Biomarkers proposed are not well described, I think. The measures section does not clarify if measures will be reported in both plasma and serum, but these can have somewhat different success rates. Notably, IL-6 and CRP can be fairly unstable and small biomarker measurement protocol variations could cause results to be uninterpretable. It would be good to be clear about the collection and storage protocols.

Response 1: We thank the reviewer for highlighting the importance of clarity in biospecimen handling. To address this, we confirm that all inflammatory biomarker analyses for (CRP, IL-6, TNF-a, SAA) and oxidative stress biomarkers (malondialdehyde, 8-isoprostane) will be conducted exclusively in serum whilst ACE2 analysis will be conducted on plasma. For serum biomarker analysis, venous blood will be collected into serum-separating tubes (BD Vacutainer® Serum Tubes), allowed to clot at room temperature for 60 minutes, and centrifuged at 1,300 × g for 10 minutes at room temperature. Serum will be aliquoted immediately into aliquots of approximately 250 µl. To prevent degradation of isoprostanes, 5mg/mL butylhydroxytoluene (BHT; Sigma-Aldrich) will be added to a single aliquot. Aliquots will then be stored at –80°C to minimise degradation and eliminate repeated freeze–thaw cycles. These procedures are consistent with best practice for cytokine and inflammatory marker preservation. Samples will be batch processed to avoid inter-assay variability in results.

Measurements will be performed blinded and independently. CRP will be assessed using high-sensitivity ELISA (detection limit, 0.35 ng/ml; Alpha Diagnostics). IL-6 will be measured using a high-sensitivity ELISA (detection limit 4.7 pg/mL; BD OptEIA ELISA). TNF-a using a high-sensitivity ELISA (detection limit 15.6 pg/mL; Invitrogen). To determine SAA levels, an ELISA will be used (detection limit, 1.1 ng/ml; Anogen). Malondialdehyde will be measured spectrophotometrically (Cell Biolabs). Isoprostane will be measured using a high-sensitivity ELISA (detection limit 1 pg/mL; Ray Biotech). All biomarkers are validated in serum and widely applied in large COPD and cognitive cohorts. Endogenous inhibitors of ACE2 activity will be removed and ACE2 catalytic activity will be measured using a validated, sensitive quenched fluorescent substrate-based assay. All samples will be assayed in duplicate with internal controls and pooled QC samples on each plate to ensure reproducibility.

We will add these details to the Methods section. We are confident that this protocol will yield stable, interpretable data and mitigate concerns regarding biomarker variability.

Comment 2: Typo in the abstract: "An prospective..." should be "A prospective..."
Response 2: We thank the reviewer for bringing this to our attention and have corrected this.

Comment 3: I found the power analysis to be confusing and maybe optimistic. In our data, for example, the correlation between CRP and IL-6 is R=0.33. A back of the napkin calculation suggests that a correlation of 0.33 is powered (using power = 0.9, a = 0.05) only in a sample with ≥95 people. It would be good for the authors to explain their expected effect sizes and what their power simulations assumed.
Response 3: Thank you — this is a helpful point. A priori power calculations were performed in G*Power v3.1.9.7. Our primary endpoint is the within-subject change in cognitive function between acute (AECOPD) and recovery timepoints. Based on prior studies reporting moderate within-subject effects in cognition in this population (Dodd et al 2013; DOI: 10.1378/chest.12-2099), we powered the paired analysis assuming a standardized mean change (Cohen's d) of 0.6. With two-tailed α = 0.05 and power = 0.90, this requires ~30 participants to detect the expected within-subject change; allowing for 20% dropout we therefore plan to recruit 40 participants (target analysed n = 32).

We note that sample size requirements for correlation analyses are different: for example, detecting a Pearson correlation of r = 0.33 with 90% power and α = 0.05 would require ~93 participants. Thus, the study is primarily powered to detect within-subject changes in cognition, and the analyses of correlations between inflammatory/oxidative markers and cognitive change are considered secondary outcomes. Correlation results will be reported with 95% confidence intervals and interpreted cautiously; the manuscript will explicitly acknowledge that the present study is not sufficiently powered to detect modest correlations (e.g., r = 0.3) with 90% power.

Comment 4: I don't know what this means: "Outcome measures will be collected during admission for AECOPD and following stabilization in the outpatient setting (approximately day 45). Day 45 was selected as participants will likely be in a stable state after recovery from

the exacerbation event." I think this means that the authors will have two time-points at study entry and at day 45? If so, the multilevel models cannot be appropriately run and a third time-point would be expected.

Response 4: We thank the reviewer for this important clarification. The study design includes two time-points per participant: (i) during admission for AECOPD (acute state) and (ii) after recovery in the outpatient setting (day 45, stable state). We agree that multilevel (mixed-effects) models are most informative when three or more repeated measures are available to model individual trajectories. With two observations per participant, the model simplifies to a paired comparison.

To address this, we have revised our analysis plan to use paired analyses as the primary approach (e.g., paired t-tests or Wilcoxon signed-rank tests for within-participant change between acute and stable states). We will also use linear regression models with time (acute vs. recovery) as a fixed effect and participant ID included as a clustering variable, allowing the incorporation of covariates such as age, sex, smoking status etc.

While additional time-points would allow modelling of longitudinal trajectories, our design is focused on characterising the acute-to-recovery transition following AECOPD, which we believe remains both clinically and biologically informative.

Comment 5: For recruitment, the authors note that "Potential participants will be identified by their treating healthcare team. If the participant is interested in obtaining more detailed information, they will be contacted by the study coordinator, who is not in a dependent relationship with the patient and will provide further information." I am not sure who judges whether the participant is interested, nor what the discussion around personal benefits to participating in research could be in the Australian situation. A lack of understanding about the incentives drawing individuals into your study could reduce generalizability of any findings so some discussion of this process could be helpful.

Response 5: We thank the reviewer for this comment and have included more detail to the recruitment section to include "Potential participants admitted with a non-infective exacerbation of COPD or following clearance from a viral exacerbation (excluding COVID-19) will first be identified by the respiratory ward medical team and provided with a patient information sheet.. If the participant is interested in obtaining more detailed information, the clinical trials nurse and principal investigator will then address any questions the patient may have regarding participation prior to obtaining written informed consent, who is not in a dependent relationship with the patient and will provide further information. Participation is voluntary and participants will not be offered any inducements to take part in the study. The study coordinator will inform the patients that their decision to participate or not will not affect their care, that their data will be held securely, and they will not be identified in any study publication. Written informed consent will be obtained by study investigators once participants have had adequate opportunity to consider their involvement and ask questions."

Comment 6: ACE2 is highly associated with COVID-19 severity and, therefore, with the presence of post-acute sequelae of COVID-19 (long-COVID or PASC). Since studies suggest that there are changes in cognition aligning directly with PASC as well, it would be good for the authors to include a COVID infection history and a PASC symptoms inventory.

Response 6: We appreciate the reviewer's suggestion regarding COVID-19 infection history and post-acute sequelae (PASC). All patients with an infective exacerbation of COPD will undergo viral PCR testing for COVID. Patients with COVID related exacerbation or PASC symptomatology will not be referred to the trial. Clinical histories will be obtained by the treating teams to identify individuals with ongoing post-COVID complications, who would therefore be excluded.

While plasma ACE2 levels have been associated with COVID-19 severity (Patel et al., Eur Respir J), our recent work (Nature Immunology) demonstrates that although ACE2 is elevated during acute COVID-19 infection, levels are not further increased in individuals with long COVID. Consequently, the potential confounding influence of prior COVID-19 infection on ACE2 levels or cognitive outcomes in this cohort is expected to be minimal.

Comment 7: Linear mixed methods are proposed for this study. These are likely to work well with some of the outcomes, but may not work well with the MMSE (which is highly abnormally distributed in most populations) and could have difficulty with some of the biomarkers (which tend to be skewed away from a their respective biological floors). The authors might consider secondary approaches in the case that they find that the longitudinal variance is abnormal.

Response 7: We thank the reviewer for highlighting this issue. With only two time-points (admission and recovery), we will primarily use paired analyses to assess within-subject changes. For normally distributed outcomes, paired t-tests will be used; for non-normal outcomes (e.g., MMSE or skewed biomarkers), we will apply Wilcoxon signed-rank tests or generalized linear models as appropriate. Covariate-adjusted analyses will use linear regression with time as a fixed effect and participant ID as a clustering variable to account for repeated measures. Log-transformations will be applied for skewed biomarkers, and sensitivity analyses will compare transformed vs. untransformed results. Effect sizes with 95% confidence intervals will be reported. This approach ensures robust, interpretable analyses across all outcomes while allowing covariate adjustment.

VERSION 2 - REVIEW

**Reviewer**	**1**
**Name**	**Clouston, Sean**
**Affiliation**	**Stony Brook University, Family, Population, and Preventive Medicine**

Date 10-Oct-2025

COI

I commend the authors on their clear and considerate efforts on this review.
